# Differential Expression of Long Noncoding RNA HOTAIR in Intestinal Metaplasia and Gastric Cancer

**DOI:** 10.14309/ctg.0000000000000483

**Published:** 2022-03-28

**Authors:** Vytenis Petkevicius, Cosima Thon, Ruta Steponaitiene, Jurgita Skieceviciene, Dainius Janciauskas, Doerthe Jechorek, Peter Malfertheiner, Juozas Kupcinskas, Alexander Link

**Affiliations:** 1 Department of Gastroenterology, Lithuanian University of Health Sciences, Kaunas, Lithuania;; 2 Institute for Digestive Research, Lithuanian University of Health Sciences, Kaunas, Lithuania;; 3 Department of Gastroenterology, Hepatology and Infectious Diseases, Otto-von-Guericke University Hospital, Magdeburg, Germany;; 4 Department of Pathological Anatomy, Lithuanian University of Health Sciences, Kaunas, Lithuania; and; 5 Institute of Pathology, Otto-von-Guericke University Hospital, Magdeburg, Germany.

## Abstract

**INTRODUCTION:**

High expression of HOTAIR promotes tumor growth and carries a dismal prognosis for the patient. We investigated the prognostic value of HOTAIR expression in gastric cancer (GC) and systematically delineate the expression in relation to *Helicobacter pylori* infection and preneoplastic changes.

**METHODS:**

HOTAIR expression was analyzed in surgical paired tissue samples of patients with GC and biopsy samples from patients with atrophic gastritis and/or intestinal metaplasia (AG ± -IM), chronic nonatrophic gastritis, and controls. The cancer genome atlas (TCGA) data were used for validation. HOTAIR expression was evaluated in sera and ascites of patients with GC. Quantitative HOTAIR expression analysis was performed using quantitative polymerase chain reaction, and LINE-1 methylation was assessed by bisulfite pyrosequencing.

**RESULTS:**

HOTAIR was more frequently detected in tumor tissues compared with adjacent gastric mucosa (65.4% vs 8.6%). HOTAIR expression was associated with depth of tumor invasion and tumor location and with shorter overall survival in patients with diffuse-type GC as confirmed in the TCGA cohort. HOTAIR was not detectable in controls but was found in 2.2% of patients with chronic nonatrophic gastritis and 18.3% of patients with AG ± IM, which was further associated with IM, grade of IM, and *H. pylori* positivity.

**DISCUSSION:**

HOTAIR expression was associated with GC and preneoplastic changes of stomach mucosa. Although HOTAIR expression was strongly linked to IM, HOTAIR expression was only associated with worse prognosis in Lauren diffuse and not intestinal type of GC. Further studies are needed to evaluate the value of HOTAIR as diagnostic and predictive biomarker in IM and translational therapeutic relevance of HOTAIR in diffuse-type GC.

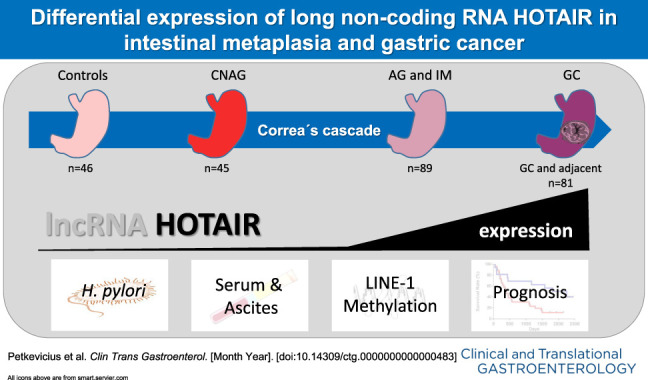

## INTRODUCTION

Gastric cancer (GC) remains the third leading cause of cancer-related death (1). Most patients are diagnosed at the advanced stage of the disease with a high mortality rate (2). Because early diagnosis and proper treatment of patients is associated with decreased mortality, the discovery of novel noninvasive biomarkers with high sensitivity and specificity is crucially needed (3). Various circulating molecules in blood, including pepsinogens (4), microRNAs (5), long noncoding RNAs (lncRNAs) (6), and circular RNAs (7), have been identified in patients with GC and are believed to contribute to improved identification of patients at risk for GC. However, at present, no specific biomarkers for preneoplastic changes or early GC have been identified so far (8).

LncRNAs regulate gene expression through various pathways that involve chromatin modification, transcription, and posttranscription processing (9). They play an important role in carcinogenesis and drug resistance in different cancer types (10). Various lncRNAs are highly expressed in GC tissues and have been evaluated as GC biomarkers (6,8). LncRNA Hox transcript antisense intergenic RNA (HOTAIR) is upregulated in GC tissue (11,12). HOTAIR targets miR-34a and activates the PI3K/AKT pathway and leads to tumor progression by inhibiting apoptosis (13). HOTAIR may promote GC cell migration and invasion through regulation of E-cadherin (14).

In clinical studies, increased HOTAIR expression was associated with advanced tumor stages, higher grades, and metastasis (11,14). Overexpression of HOTAIR was significantly associated with unfavorable prognostic outcomes in patients with GC, although others failed to confirm its prognostic role (15). Elevated HOTAIR expression was linked to peritoneal dissemination in GC, and small interfering RNA knockdown of HOTAIR led to inhibition of cell proliferation, migration, and invasion *in vitro* and *in vivo* models (16).

The available data related to HOTAIR in GC originate mostly from the Asian population, the potential prognostic role in European population has not been studied yet. Furthermore, although HOTAIR has been the focus of studies conducted in advanced-stage GC, its role in preneoplastic stages remains unexplored. Therefore, our study aimed to determine and characterize the HOTAIR expression in GC and along the progression of preneoplastic gastric changes and to assess the clinicopathological and prognostic value of HOTAIR in patients with GC. In addition, we performed a series of complimentary analyses to explore the relation of HOTAIR to global LINE-1 methylation in GC.

## METHODS

### Ethics approval and consent to participate

The study was a part of the ERA-Net PathoGenoMics project, and the Institutional Review Board of Otto-von-Guericke University Magdeburg approved the study protocol Nr. 80/2011. The Kaunas Regional Bioethics Committee has also approved the samples collection Nr. BE-2-10. The ascites samples were obtained at the Department of Gastroenterology, Hepatology and Infectious Diseases at Otto-von-Guericke University Magdeburg (Approval Nr. 85/2010). Written informed consent was obtained from all patients.

### Sample collection

Patients with GC were recruited in the Departments of Gastroenterology and Surgery at the Hospital of Lithuanian University of Health Sciences in Kaunas (Lithuania) between 2010 and 2013. Patients with non-neoplastic mucosa were recruited in the Department of Gastroenterology, Hepatology and Infectious Diseases at Otto-von-Guericke University Magdeburg (Germany). The study material included 81 GC tumor tissue samples (T-GC) with paired adjacent nontumorous gastric mucosa samples (NT-GC), 46 control (N) tissue samples of patients with histologically confirmed normal gastric mucosa, 46 tissue samples from patients with chronic nonatrophic gastritis (CNAG) without intestinal metaplasia (IM), 109 tissue samples with AG and/or IM, 23 GC serum samples, and 45 ascites samples from patients with peritoneal carcinomatosis from various tumors including GC. All patients were of European descent. The detailed information on the GC cohort has been reported in our previous studies (17,18). Briefly, all subjects underwent primary surgery without prior neoadjuvant therapy. The characterization of patients with GC regarding HOTAIR positivity is shown in Table 1. Controls (N) and patients with preneoplastic changes (CNAG, AG and/or IM) were referred for upper GI endoscopy, and antrum biopsies were obtained for the further molecular analysis. Detailed inclusion and exclusion criteria are reported elsewhere (19,20). Characterization of patients with preneoplastic conditions is presented in Table 2. Specimens from patients with GC were prospectively collected after surgical resection and histopathologically confirmed as gastric adenocarcinoma. Classification of GC was based on the Lauren criteria. Histological characterization of gastritis was performed according to the updated Sydney classification (21). The status of *Helicobacter pylori* for controls and patients with preneoplastic changes was determined by serology, microbiology, and histology as previously described (22). Tissues or biopsies were snap frozen in liquid nitrogen and stored at −80 °C until analysis.

**Table 1.** Clinicopathological features of patients with gastric cancer in relation to HOTAIR positivity

	HOTAIR positive, n = 53 (65.4%)		HOTAIR negative, n = 28 (34.6%)		*P* Value
	N	Proportion	n	Proportion	
Age, yr, mean ± SD	67.2 ± 10.9		63.3 ± 12.8		0.151^a^
Sex					
Male	29	61.7%	18	38.3%	0.407^b^
Female	24	70.6%	10	29.4%	
Tumor localization					
Cardia	8	100.0%	0	0.0%	**0.016** ^b^
Corpus	24	53.3%	21	46.7%	
Antrum	21	75.0%	7	25.0%	
UICC classification					
I	7	43.8%	9	56.2%	0.237^b^
II	15	71.4%	6	28.6%	
III	25	69.4%	11	30.6%	
IV	6	75.0%	2	25.0%	
T					
1 + 2	8	44.4%	10	55.6%	**0.034** ^b^
3 + 4	45	71.4%	18	28.6%	
N					
0	15	51.7%	14	48.3%	0.147^b^
1	12	80.0%	3	20.0%	
2	7	53.8%	6	46.2%	
3	18	78.3%	5	21.7%	
Unknown	1	100.0%	0	0.0%	
M					
0	46	63.9%	26	36.1%	0.629^b^
1	6	75.0%	2	25.0%	
Unknown	1	100.0%	0	0.0%	
Grading					
1	1	33.3%	2	66.7%	0.466^b^
2	20	69.0%	9	31.0%	
3	32	65.3%	17	34.7%	
Laurén classification					
Diffuse type	26	59.1%	18	40.9%	0.366^b^
Intestinal type	18	69.2%	8	30.8%	
Mixed type	5	71.4%	2	28.6%	
Unknown	4	100.0%	0	0.0%	

HOTAIR positivity was defined by the cutoff of ≤40.

a Student *t* test.

b χ^2^ test.

M, metastasis; N, controls; T, tumor; UICC, Union for International Cancer Control.

**Table 2.** Characterization of patients with HOTAIR-positive and -negative and preneoplastic changes

	HOTAIR positive		HOTAIR negative		*P* Value
	n	Proportion	n	Proportion	
Group					
Control	0	0.0%	46	100.0%	**0.0030**
CNAG	1	2.2%	45	97.8%	
AG ± IM	20	18.3%	89	81.7%	
IM					
No IM	4	2.9%	134	97.1%	**<0.0001**
IM (total)	17	27.0%	46	73.0%	
Mild IM	7	18.0%	32	82.0%	**0.0278**
Moderate IM	4	28.6%	10	71.4%	
Severe IM	6	60.0%	4	40.0%	
*H. pylori*					
*H. pylori*−	7	7.1%	91	92,9%	
*H. pylori*+ (total)	14	13.6%	89	86.4%	
*H. pylori*+ (serology)	4	15.4%	22	84.6%	
*H. pylori*+ (histology and/or microbiology)	10	13.0%	67	87.0%	
IM and *H. pylori* groups					
IM− *H. pylori*−	2	2.9%	67	97.1%	**<0.0001**
IM− *H. pylori*+	2	2.8%	69	97.2%	
IM+ *H. pylori*−	5	17.2%	24	82.8%	
IM+ *H. pylori*+	12	35.3%	22	64.7%	

HOTAIR positivity was defined by the cutoff of ≤40.

AG, atrophic gastritis; CNAG, chronic nonatrophic gastritis; IM, intestinal metaplasia.

### RNA isolation, reverse transcription, and quantitative real-time polymerase chain reaction

Total RNA from frozen tissue samples was extracted using the commercial RNeasy Plus Universal Mini Kit (QIAGEN, Valencia, CA) following the manufacturer's recommendations with minor modifications as described previously (17). Quantitative and qualitative analysis of RNA samples was performed spectrophotometrically and by gel electrophoresis. For quantitative real-time polymerase chain reaction (qRT‐PCR), cDNAs from all samples were synthesized from 1 μg of total RNA. Quantitative HOTAIR real-time PCR was performed using the BioRad CFX Cycler System (BioRad, Hercules, CA) in duplicate for each sample. The housekeeping β-actin gene was used for normalization. The quality of the PCR reaction was confirmed by no template controls and reference samples and specificity by melting curve analyses. The relative quantification was calculated by the 2^−ΔCT^ method. The cycle threshold value of ≤40 was classified as detectable.

### DNA isolation and methylation analysis

DNA was extracted from the same tissue samples pretreated with QIAzol Lysis reagent and chloroform according to the manufacturer's protocol (provided by QIAGEN). Purified genomic DNA was bisulfite modified using the Cells-to-CpGTM Bisulfite Conversion Kit (Life Technologies, Carlsbad, CA) following the manufacturer's protocol, as described previously (17,18). Briefly, quantitative methylation analyses of long interspersed nucleotide element 1 (LINE-1) were performed by bisulfite pyrosequencing on PyroMark Q96 ID (QIAGEN) using PyroMark Gold Q96 reagents (QIAGEN). For further quantitative methylation analysis, we used the mean methylation level of analyzed CpG sites.

### Survival analysis

The data on survival of patients with GC were obtained from the Lithuanian Cancer Registry and from medical records at the Hospital of Lithuanian University of Health Sciences Kaunas Clinics. Overall survival time was defined as the time from GC diagnosis to death from any cause or until the end of follow-up with a maximum of 2,500 days. Deaths up to February 28, 2017, were included in the analysis. Patients who died within 30 days after surgery were excluded from prognostic analysis to exclude potential bias through GC-unrelated cause of death. Validation of survival analysis was performed using TCGA data set for GC. For this purpose, we used the Kaplan-Meier Plotter analysis tool (http://kmplot.com/analysis/), which incorporates multiple GEO data sets for prediction of survival and prognosis (23). The survival data for HOTAIR were plotted using upper tertial as a cutoff for GC samples, similar cutoff was used for GC according to the Lauren intestinal and diffused type.

### Statistical analysis

Quantitative data are shown as mean ± SD. Mean values of age were compared using the Student *t* test. Categorical data are presented as proportions and compared using the χ^2^ test. The Wilcoxon test was used to compare paired groups. The Mann-Whitney *U* test and the Kruskal-Wallis multiple comparison test were used for unpaired analyses. Linear regression analysis was used for the association between HOTAIR expression in T-GC and NT-GC group as well as for the association between HOTAIR expression and methylation. Survival data were analyzed by the Kaplan-Meier methods and evaluated by the log-rank test. To estimate the significance of various factors that might influence the survival of patients with diffuse type of GS, univariable and multivariable Cox regression analyses were performed.

All statistical analyses were performed using the statistical package IBM SPSS Statistics for Windows, Version 22.0 (IBM, Armonk, NY) and GraphPad Prism 7.0 statistical software (San Diego, CA). A *P* value less than 0.05 was considered statistically significant.

## RESULTS

### HOTAIR in GC tissues

**Figure 1.**
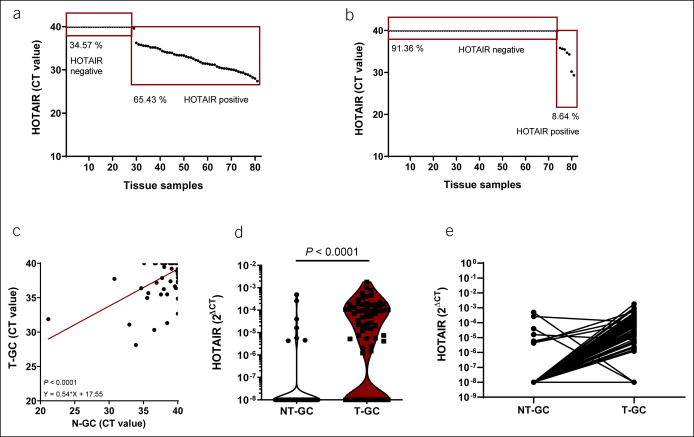
HOTAIR in patients with GC. (**a**) Global level of HOTAIR in tumorous GC tissues (T-GC, n = 81). (**b**) Global level of HOTAIR in nontumorous GC tissues (NT-GC, n = 81). (**c**) Correlation between HOTAIR levels in NT-GC and T-GC (n = 81, *P* < 0.001). (**d** and **e**) Paired levels of HOTAIR between N-GC and T-GC (n = 81, *P* < 0.001). Data presented as CT value and 2^ΔCT^. The Wilcoxon test was used to compare paired groups and linear regression analysis for association between HOTAIR positivity in N-GC and T-GC. GC, gastric cancer.

HOTAIR was more frequently present in tumor tissues (T-GC) as compared with matched adjacent nontumor (NT-GC) tissues. HOTAIR expression was detectable in 65.4% (53/81) of samples from T-GC and in 8.6% (7/81) of samples from NT-GC (Figure 1a,b). There was a statistically significant correlation for positivity between NT-GC and T-GC (*P* < 0.0001) (Figure 1c). Paired HOTAIR analysis revealed that most patients with GC had higher HOTAIR expression in T-GC tissues, whereas only 2 patients had lower expression in T-GC in comparison with NT-GC tissues (Figure 1d,e).

### HOTAIR and clinicopathological GC characteristics

Comparison of HOTAIR-positive and -negative groups according to sex, tumor localization, Union for International Cancer Control stages, TNM classification, grading, and Lauren classification is reported in Table 1. Interestingly, all tumor samples from cardia were HOTAIR positive, whereas in GC tumors from the corpus, the HOTAIR expression was found only in 53.3% (*P* = 0.016). HOTAIR expression was associated with depth of tumor invasion (T) with a positivity of 71.4% in T3-4 and 44.4% in T1-2 tumors (*P* = 0.034). There was no association with other clinicopathological GC parameters.

### Prognostic role of HOTAIR for patients with GC

**Figure 2.**
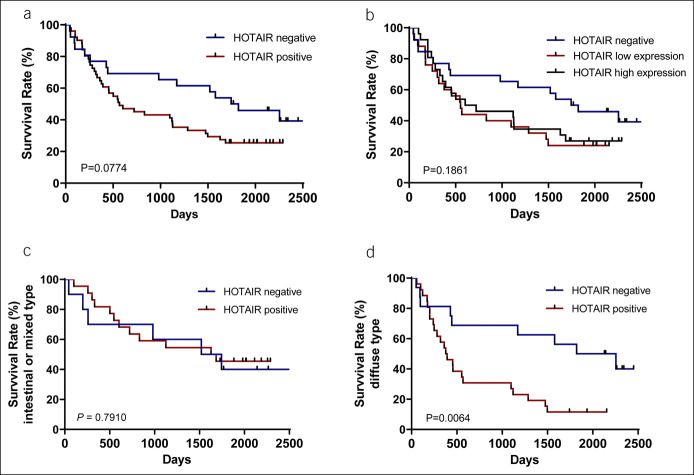
Survival rates of patients with GC depending on HOTAIR status. (**a**) All patients with GC (n = 77, *P* = 0.0774). (**b**) All patients with GC, HOTAIR-positive patients are divided into 2 groups by mean of expression level (n = 77, *P* = 0.1861). (**c**) Patients with GC with intestinal or mixed type according to the Lauren classification (n = 32, *P* = 0.7910). (**d**) Patients with GC with Lauren diffuse type (n = 42, *P* = 0.0064). Survival data were evaluated using Kaplan-Meier analyses. GC, gastric cancer.

Survival data were obtained for all subjects with GC for a period of up to 2,500 days. Four patients were excluded from analysis due to death within the first 30 days (2 with HOTAIR-positive and 2 with HOTAIR-negative T-GC samples). The median survival of 77 patients was 1,098 days. Tumor positivity for HOTAIR expression was associated with a statistical trend for a shorter overall survival of patients with GC compared with patients without detectable HOTAIR expression; however, the difference did not reach statistical significance (567 days vs 1784 days, *P* = 0.077) (Figure 2a). We then investigated whether tumors with low and high HOTAIR expression defined by the median would have phenotypical differences; however, splitting the HOTAIR-positive T-GC group into low and high HOTAIR expression did not show statistically different survival data (*P* = 0.186) (Figure 2b). Subsequently, the prognostic differences were evaluated for GC based on the Lauren classification. Survival analysis of patients with HOTAIR-positive vs HOTAIR-negative T-GC showed no difference in intestinal and mixed-type tumors (*P* = 0.79) (Figure 2c). Interestingly, patients with Lauren diffuse-type GC with HOTAIR-positive tumors had significantly worse overall survival compared with the HOTAIR-negative group (385 days vs 2039 days, *P* = 0.006) (Figure 2d). To confirm those results, we performed validation analysis on association between HOTAIR expression and prognosis in TCGA data set for GC (n = 611). As shown in Supplementary Figure S1 (http://links.lww.com/CTG/A790), patients with high expression of HOTAIR had comparable survival probability as patients with low HOTAIR expression (*P* = 0.21). In subgroup analysis, no difference was observed in patients with Lauren intestinal type (*P* = 0.73), whereas patients with diffuse type of GC show significantly worse prognosis in subjects with high HOTAIR expression (*P* = 0.032). Overall, the prognostic analysis of both cohorts suggests that high HOTAIR expression may be associated with worse prognosis specifically in subjects with Lauren diffuse-type GC, although the exact mechanistic explanation or potential cofactors (for example early recurrence, metastasis pattern, or insufficient response to chemotherapy) remain to be determined.

In univariable Cox regression analyses, only HOTAIR positivity (*P* = 0.009) and depth of tumor invasion (T) (*P* = 0.02) affected the overall survival of patients statistically significant (Table 3). Multivariable analyses performed using Cox proportional hazards model showed that HOTAIR positivity (*P* = 0.029) and age (*P* = 0.026) can independently predict the overall survival of patients with diffuse-type GC.

**Table 3.** Univariable and multivariable Cox regression analysis of overall survival in patients with diffuse-type gastric cancer

Variables	Univariable analysis		Multivariable analysis	
	HR (95% CI)	*P* Value	HR (95% CI)	*P* Value
Sex (male vs female)	1.41 (0.70–2.87)	0.336	0.65 (0.26–1.62)	0.355
Age groups (≥70 vs < 70)	2.99 (0.95–4.15)	0.068	2.75 (1.13–6.72)	**0.026**
HOTAIR positivity (positive vs negative)	3.04 (1.32–6.98)	**0.009**	3.13 (1.12–8.75)	**0.029**
Tumor localization				
Cardia vs antrum	2.40 (0.51–11.36)	0.269	2.42 (0.30–19.68)	0.409
Corpus vs antrum	1.01(0.48–2.14)	0.970	1.60 (0.64–4.04)	0.317
UICC Classification (III + IV vs I + II)	1.18 (0.58–2.39)	0.652	0.58 (0.13–2.57)	0.468
T (3 + 4 vs 1 + 2)	4.20 (1.26–14.01)	**0.020**	2.76 (0.67–11.27)	0.158
N (2 + 3 vs 0 + 1)	1.05 (0.52–2.12)	0.893	1.25 (0.30–5.29)	0.759
M (1 vs 0)	1.21 (0.365–4.00)	0.756	1.06 (0.24–4.72)	0.942
Grading (3 vs 2)	1.78 (0.621–5.12)	0.283	2.67 (0.62–11.41)	0.187

CI, confidence interval; HR, hazard ratio; M, metastasis; N, controls; T, tumor; UICC, Union for International Cancer Control.

### HOTAIR in preneoplastic changes

Having shown the clinicopathological relevance of HOTAIR in GC, we examined whether HOTAIR might be an early event of gastric carcinogenesis. To answer this question, we focused on preneoplastic changes including N, CNAG, AG ± IM samples. In addition, we looked at *H. pylori* status in controls and preneoplastic changes, as well as IM in CNAG and AG groups.

**Figure 3.**
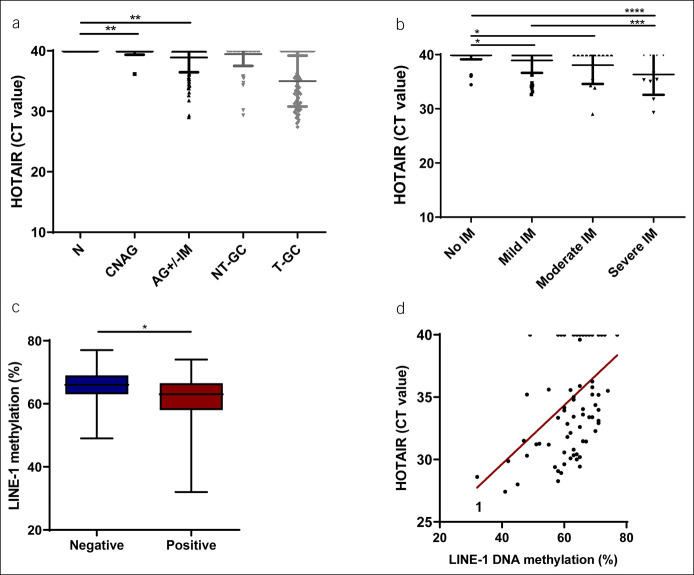
Detectability of HOTAIR in preneoplastic changes and correlation between HOTAIR and LINE-1 methylation in patients with GC. (**a**) HOTAIR in healthy (N, n = 46), nonatrophic chronic gastritis (CNAG, n = 46), atrophic gastritis and/or intestinal metaplasia (AG ± IM, n = 109), nontumorous tissues (NT-GC, n = 81), and tumorous tissues (T-GC, n = 81) in GC, **P < 0.01. (**b**) HOTAIR in mild intestinal metaplasia (IM, n = 39), moderate IM (n = 14), severe IM (n = 10), and patients with no signs of IM (n = 138), *P < 0.05, ***P < 0.001, and ****P < 0.0001. (**c**) LINE-1 DNA methylation differences between HOTAIR-positive (n = 53) and -negative (n = 27) T-GC (*P* = 0.0241). (**d**) Association between LINE-1 DNA methylation and HOTAIR in T-GC (n = 80, *P* = 0.0001). Data are presented as 2^ΔCT^. The Mann-Whitney test was used for comparison of 2 groups, the Kruskal-Wallis test for comparison of multiple groups, and linear regression analysis for association. GC, gastric cancer.

HOTAIR was undetectable in controls, although it was found in 2.2% (1/46) of CNAG and in 18.3% (20/109) of patients with AG and/or IM (Figure 3a). HOTAIR was expressed in 27% (17/63) of cases with IM, and the expression was positively associated with a higher grade of IM (*P* = 0.0278) (Figure 3b). *H. pylori* infection was statistically not more abundant in the HOTAIR-positive group (Table 2). Seven (7.1%, 1/98) patients without evidence for *H. pylori* infection had positive HOTAIR expression, and 13.6% of *H. pylori*–positive subjects showed HOTAIR positivity. The highest prevalence of HOTAIR positivity (35.3%) was found in the group of IM and *H. pylori* infection (Table 2).

### Correlation of HOTAIR with global methylation patter

Because global hypomethylation is a frequent event in GC, we considered whether HOTAIR expression might be related to epigenetic alterations. To investigate this, we compared the LINE-1 methylation in GC tumors based on HOTAIR positivity. LINE-1 methylation was significantly lower in HOTAIR-positive compared with HOTAIR-negative T-GC samples (*P* = 0.0241) (Figure 3c). Moreover, we observed significant correlation between LINE-1 hypomethylation and HOTAIR expression (*P* < 0.001) (Figure 3d), suggesting a possible link between these molecular events.

### HOTAIR in serum and in peritoneal carcinomatosis

To investigate the potential translational clinical value of HOTAIR as a noninvasive diagnostic biomarker in GC, we analyzed circulating HOTAIR levels in sera of patients with GC and in ascites samples from patients with peritoneal carcinomatosis including GC. However, we observed no reproducibly detectable HOTAIR levels in sera samples of patients with GC (n = 23) or in ascites samples of patients with peritoneal carcinomatosis from various tumors irrespective of the cancer origin (n = 45).

## DISCUSSION

In this work, we evaluated HOTAIR expression in a European cohort of patients with GC and studied its expression in gastric mucosa in relation to progression of preneoplastic changes. We found that an increased HOTAIR expression was significantly higher in GC tissues compared with the adjacent nontumorous gastric mucosa and confirmed previous findings from the Asian population (24,25). A notable finding in our work was an association between HOTAIR expression and the depth of tumor invasion. HOTAIR expression was also associated with worse overall survival in patients with the histological diffuse-type of GC. Furthermore and of the most translational relevance is that HOTAIR expression was strongly associated with IM, which was furthermore highest in *H. pylori*–positive patients.

In common with previous studies, we demonstrate a consistent HOTAIR expression in GC. However, the link between upregulation of HOTAIR in GC and different clinicopathological features varies between the studies. Some studies found that HOTAIR positivity was associated with lymph node metastasis and higher TNM stage (14,24,26). Li et al. (25) and Liu et al. (27) additionally showed a link between HOTAIR expression and the depth of tumor invasion, differentiation, and distant metastasis. Based on our results, HOTAIR positivity was associated neither with lymph node nor with distant metastasis but was associated with the depth of invasion.

Recent meta-analysis summarizing the available prognostic data indicates that high expression level of lncRNA HOTAIR is associated with poor overall survival in patients with GC (15). The data from our European cohort confirmed the prognostic value of HOTAIR in GC; however, this was true only for GC of Lauren diffuse type, which was further confirmed using the data from TCGA cohort. Our findings are in line with Endo et al. data where the authors indicated that the upregulation of HOTAIR predicted a poor prognosis only in patients with diffuse-type GC (28). Liu et al. have also compared expression levels of HOTAIR in diffuse and intestinal-type GC and found that expression was significantly higher in diffuse-type GC, and the worst prognosis was observed in diffuse but not intestinal-type GC with high HOTAIR expression (29). These data clearly suggest a unique functional role of HOTAIR in different Lauren GC types.

Although the clinical value of HOTAIR expression has been investigated in several studies before, the significance of HOTAIR expression in preneoplastic changes of stomach mucosa has not received sufficient evaluation. To better characterize the HOTAIR role in GC carcinogenesis, we analyzed HOTAIR expression in preneoplastic changes. Zhang et al. reported on initial HOTAIR expression analysis in gastric tissues, showing the highest expression in AG (12). In our results, we observed no expression of HOTAIR in controls, but it was upregulated in patients with CNAG and AG and/or patients with IM. Further detailed evaluation revealed that HOTAIR positivity was strongly related to IM and was positively associated with the severity of IM. This observation suggests that certain molecular events during Correa cascade of carcinogenesis specifically trigger HOTAIR activation. In addition, we looked at *H. pylori* status in both controls and preneoplastic changes and identified that HOTAIR positivity was most prevalent in patients with IM infected with *H. pylori*. Stepwise increase of HOTAIR expression from AG/IM to T-GC indicates further that HOTAIR is a unique molecular event that is likely associated with the point of no return and may be associated with the risk of progression from preneoplastic changes to GC. Further long-term studies are needed to confirm whether HOTAIR positivity may be used as a molecular marker for IM and as the most reliable marker for preneoplastic changes in gastric mucosa.

Alterations in DNA methylation play an important role in the carcinogenesis of GC in multiple ways, including genomic instability (30). Global DNA hypomethylation refers to a decrease in DNA methylation across the entire genome. Long interspersed element 1 (LINE-1) methylation may be used as a surrogate marker of global DNA methylation (31). To further illuminate on the molecular events in gastric carcinogenesis related to HOTAIR, we correlated the HOTAIR expression with LINE-1 methylation. The strong negative correlation between LINE-1 methylation and HOTAIR expression in T-GC samples suggests potential involvement of DNA methylation in regulation of HOTAIR expression or the opposite effect of HOTAIR on global DNA methylation.

Having shown the high expression of HOTAIR in GC and IM, we considered whether HOTAIR might be used as a noninvasive biomarker for cancer detection. Several studies have recently demonstrated that lncRNAs are detectable in the plasma of patients with cancer and, therefore, may be used as noninvasive biomarkers for cancer detection (3,8). Elsayed et al. found that the plasma level of HOTAIR was significantly higher in patients with GC compared with healthy controls. They concluded that plasma HOTAIR could diagnose GC with 88% sensitivity and 84% specificity (11). On the contrary, Arita et al. did not identify a significant difference in the plasma level of HOTAIR between patients with GC and controls (32). Remarkably, we performed repeated analysis to identify HOTAIR in sera samples of patients with GC, but no reproducible HOTAIR levels were detectable. In similar fashion, although noncoding RNA, specifically miRNAs, can be identified in peritoneal fluid and ascites from patients with cancer with peritoneal carcinomatosis (33), we measured no detectable HOTAIR levels in patients with peritoneal carcinomatosis, including patients with GC.

Over the past few years, substantial effort has been made to identify specific biomarkers for diagnosis, prediction of cancer development, and prediction of therapy response. Despite very intriguing results, there are also several limitations that need to be addressed in further studies. Specifically, we were able to evaluate the prognostic value of HOTAIR in patients with GC, but the prediction of chemotherapy or the risk of disease recurrence has not yet been assessed. This cohort included only therapy-naive patients and further studies take to consideration neoadjuvant or palliative settings are needed. In addition, we have no data on differences in subtypes of GC including molecular TCGA classification or hereditary syndromes or even pernicious anemia. Furthermore, we used a targeted approach based on the data from GC tumor tissues, but in future studies, a multiomics approach may provide additional insight into the concomitant changes related to HOTAIR expression. The most striking data of our work relate specific HOTAIR expression in IM and merit further in-depth analysis and correlation to GC progression. In this view, additional analysis taken to account different subtypes of IM (complete vs incomplete) may be helpful to further elucidate the link. Finally, mechanistic studies are need to explain the fact that although HOTAIR seems to be associated with progression of Correa cascade as it is expressed initially in IM, it seems to have no prognostic impact in Lauren intestinal-type GC (34). It may be possible that HOTAIR expression in diffuse-type GC may trigger additional mechanisms responsible for tumor progression or be triggered by microbial community for instance *Fusobacterium nucleatum* (20,35).

In summary, our data provide novel evidence suggesting an early involvement of HOTAIR in gastric carcinogenesis. Although the prognostic potential of HOTAIR in GC has been reported before, our analysis allowed for the first time to link the HOTAIR expression with IM in gastric mucosa. This may lead to the development of molecular biomarkers that indicate premalignant changes predictive for the cascade of gastric carcinogenesis and advance surveillance strategies.

## CONFLICTS OF INTEREST

**Guarantor of the article:** Alexander Link, MD, PhD.

**Specific author contributions:** V.P., C.T., and R.S. performed the experiments. J.S., D.J., P.M., J.K., and A.L. provided clinical material and resources. V.P., C.T., and A.L. performed data analysis and drafting of the manuscript. J.K. and A.L. study concept and design. A.L.: supervision and project administration. All authors reviewed and approved the final version of the manuscript.

**Financial support:** Sample collection was supported by the BMBF Nr. BMBF-0315905D in the frame of ERA-NET PathoGenoMics to P.M. A.L. is supported by the funds of European Commission through the “European funds for regional development” (EFRE) as well as by the Ministry of Economy, Science and Digitalization of Saxony-Anhalt as part of the“Autonomy in old Age“(AiA) research group for“LiLife”Project (Project ID: ZS/2018/11/95324). J.S. and J.K. are supported European Social Fund (project No 09.3.3-LMT-K-712-01-0130) under grant agreement with the Research Council of Lithuania (LMTLT) for MULTIOMICS project.

**Potential competing interests:** None to report.

## Supplementary Material

SUPPLEMENTARY MATERIAL
