# Supplementary material for: Differential Expression of Long Noncoding RNA HOTAIR in Intestinal Metaplasia and Gastric Cancer
Source: Clin Transl Gastroenterol. 2022 Mar 28;13(5):e00483. doi: 10.14309/ctg.0000000000000483 (PMC9132515; doi:10.14309/ctg.0000000000000483)

Supplementary Figure S1. Probability of survival based on HOTAIR expression in TCGA cohort of GC patients. (A) All GC patients (n=611, p=0.21). (B) Lauren´s intestinal type GC patients, HOTAIR-positive patients are divided into two groups by mean of expression level (n=257, p=0.73). (C) Lauren´s diffuse type GC patients (n=233, p=0.0032). Survival data were performed using Kaplan-Meier Plotter analysis tool (http://kmplot.com/analysis/).

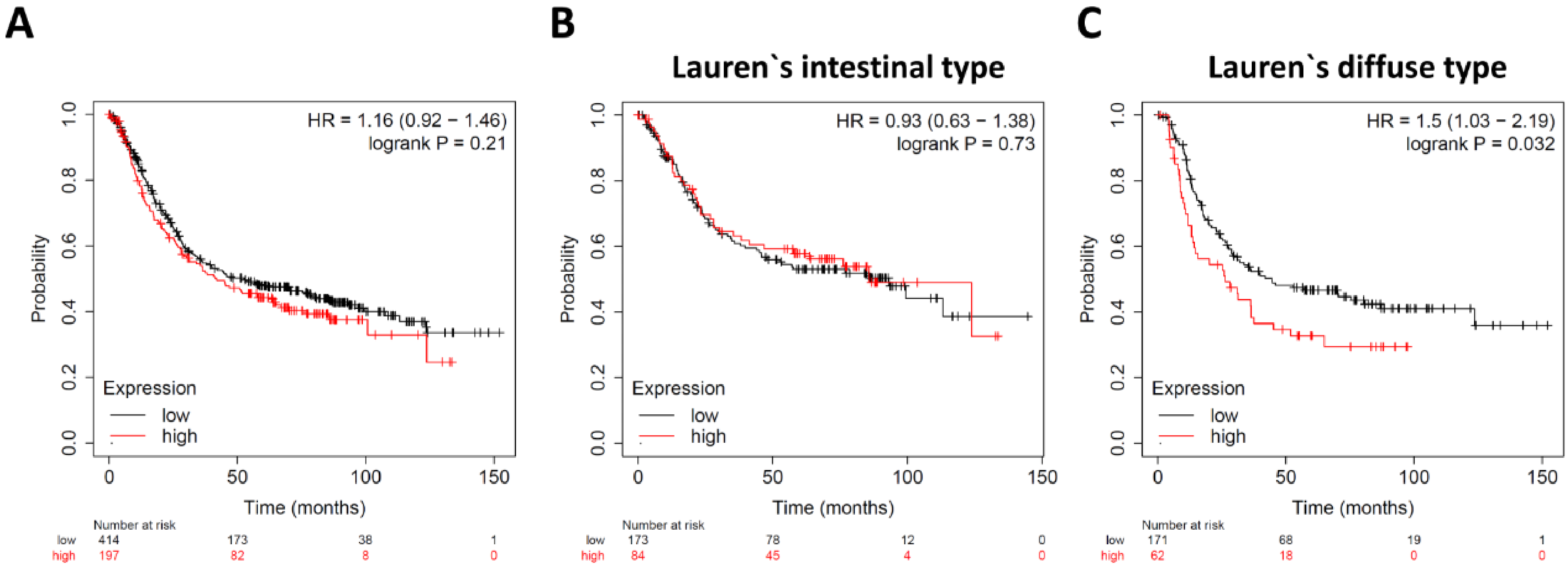

Supplement: SUPPLEMENTARY MATERIAL [file ct9-13-e00483-s002.pdf]
